# Suicide following hospital admission for mental health conditions, physical illness, injury and intentional self-harm in Victoria, Australia

**DOI:** 10.1371/journal.pone.0271341

**Published:** 2022-07-11

**Authors:** Dasamal Tharanga Fernando, Angela Clapperton, Janneke Berecki-Gisolf

**Affiliations:** Victorian Injury Surveillance Unit, Monash University Accident Research Centre, Monash University, Clayton, Victoria, Australia; Peking University, Institute of Mental Health, CHINA

## Abstract

**Objective:**

The majority of suicide decedents have had contact with health services close to their death. Some of these contacts include admissions to hospitals for physical and mental health conditions, injury and intentional self-harm. This study aims to establish and quantify the risks of suicide following hospital admission for a range of mental and physical illnesses.

**Methods:**

A retrospective analysis was carried out on existing morbidity and mortality data in Victoria. Data was extracted from the Victorian Admitted Episodes Dataset and the Victorian Suicide Register. Unplanned hospital admissions among adult patients (> = 15 years of age), discharged between 01 January 2011 and 31 December 2016 (2,430,154 admissions), were selected. Standardised Mortality Ratios were calculated for conditions with at least five linked suicides within one year of discharge from hospital.

**Results:**

Forty-three conditions defined at the three-digit level of the International Statistical Classification of Diseases and Related Health Problems 10th Revision, were associated with at least five subsequent suicides (within one year of hospital discharge); 14 physical illnesses, 5 symptoms, signs and abnormal clinical and laboratory findings, 12 mental health conditions, and 12 types of injury and poisonings. The highest Standardised Mortality Ratios were for poisonings (range; 27.8 to 140.0) and intentional self-harm (78.8), followed by mental health conditions (range; 15.5 to 72.9), symptoms, signs and abnormal clinical and laboratory findings (range; 1.4 to 43.2) and physical illnesses (range; 0.7 to 4.9).

**Conclusions:**

Hospital admissions related to mental health conditions and injury and poisonings including self-harm were associated with a greater risk of suicide than physical conditions. Mental health conditions such as depressive episodes, personality disorders and psychotic episodes, injuries caused by intentional-self-harm and poisonings by certain types of drugs, carbon monoxide and hormones such as insulin can be prioritised for targeting suicide prevention initiatives for persons discharged from hospitals.

## Introduction

Suicide is a major public health concern globally, with an estimated 800,000 suicides occurring worldwide every year [1]. In 2018, there were 3,046 suicides registered in Australia which relates to 8 deaths per day [2]. Recent statistics published by the Coroner’s Court of Victoria showed that there were 2,787 suicides in the state of Victoria between 2016 and 2019, which averages to 697 suicides per year [3].

Studies in Western, developed countries have consistently shown that a high proportion of people who die by suicide have a diagnosed, or diagnosable mental health condition at the time of death [4–7]. In addition to mental health conditions, physical illness has consistently been associated with suicide. A recent study found that physical illness was indicated as the main reason for suicide in almost 20% of cases [8], and another study has shown a link between hospital admissions for constipation, gastritis, diabetes, alcoholic liver disease, pneumonia and urinary tract infections and increased risk of suicide [9]. Studies have also shown that certain injuries such as traumatic brain injury are likely to increase the risk of suicide [10, 11], and non-fatal suicide attempts are also strongly correlated with subsequent suicide [12–14].

The majority of people who die by suicide have had contact with health services (i.e., general practitioners, outpatient or inpatient services) proximal to their death [15–20]. These contacts represent potential suicide prevention opportunities [16], through identification of persons at risk of suicide, provision of treatment and/or delivery of interventions.

The main aim of this study is to establish and quantify risks of suicide following hospital admissions for a range of mental health conditions, physical illnesses, injury and intentional self-harm in Victoria.

## Methods

### Study design

A retrospective analysis of existing morbidity and mortality data in the state of Victoria, Australia was carried out for this study.

### Data sources and linkage

Morbidity data was extracted from the Victorian Admitted Episodes Dataset (VAED) and mortality data was extracted from the Victorian Suicide Register (VSR) and the Victorian Death Index (VDI).

The VAED is a unit record data file maintained by the Department of Health and Human Services on all public and private hospital admissions in Victoria, which consists of patient demographics and morbidity information. Morbidity information includes 40 fields related to disease, injury and external causes, coded to the International Statistical Classification of Diseases and Related Health Problems Tenth Revision, Australian Modifications (ICD-10-AM) [21].

The VSR is an ongoing register established by the Coroners Court of Victoria and contains information on all suspected and coroner-determined suicides reported to the Victorian Coroners Court [22].

The VDI contains all death-records for Victoria, and is obtained from the Registrar of Births, Deaths and Marriages in Victoria.

Data provision and linkage was undertaken by the Centre for Victorian Data Linkage (CVDL). Data linkage performed by CVDL used patient-specific identifiers and deterministic data linkage. The Coroners Court of Victoria (CCOV) supplied VSR data to CVDL via secure data exchange portals. CVDL linked the VSR data with the VAED and VDI, and all data were de-identified. The de-identified datasets, containing a linkage identification number, were transferred to researchers at Monash University using the secure data exchange portal.

### Study population

#### Inclusion criteria

The study population consisted of adults (> = 15 years of age) with unplanned hospital admissions in Victoria, Australia, discharged between 01 January 2011 and 31 December 2016 (3,640,529 episodes) as recorded in the VAED. They were followed up until 31 December 2017 in the VSR and VDI. Consecutive records of inward transfers from other hospitals or statistical separations (changes in care type) within the same hospital were considered to be part of one period of care (POC). All initial POCs, that is the POC that included the index (initial) admission for each patient were retained for final analysis. In cases where the patient was newly admitted subsequent to the initial POC, these records were also retained and included as unique POCs, provided the subsequent admission took place at least one-year post discharge of the previous admission.

#### Exclusion criteria

Planned admissions were excluded as some of these were for diagnostic investigations (which are not always indicative of the presence of a particular disease), and the rest were ‘elective’ medical or surgical treatment. These also include kidney patients on routine dialysis treatments (with multiple admissions) and cancer patients on chemo therapy etc. (with multiple admissions), which does not allow for ideal incidence estimation. The following were also excluded: POCs ending with a death in hospital (because the outcome-of-interest was death following discharge), hospital admissions of intersex people (to preserve confidentiality of small cell counts: recorded intersex status was rare), admissions with missing principal diagnosis codes (205 admissions) and admissions with unrealistic time to death (i.e., admission dates occurring after the date of death: 951 records (0.03%), which could be due to data linkage inaccuracy). The final number of episodes retained for the analysis was 2,430,154.

### Outcome measure

The study outcome measure was suicide within one year of hospital discharge. The rationale for starting the 1-year follow-up at discharge (and not at admission) was to capture only post-discharge exposure: suicide during the hospital admission was therefore excluded.

### Selection of conditions

The conditions considered for this study were physical illnesses (acute and/or chronic); injury; symptoms, signs and abnormal clinical and laboratory findings; and mental health conditions. All conditions were analysed at the three-digit level in the ICD-10-AM ranging from A00-T79 appearing in the principal diagnosis field at admission. Conditions were included if they were associated with at least five suicides within one year of discharge from hospital (43 conditions fulfilled these criteria).

Due to known associations between intentional-self harm and subsequent suicide, admissions for intentional self-harm were also examined. Intentional self-harm admissions were selected if the external cause codes "X60" to "X84" (intentional self-harm) appeared in *any* of the 40 diagnosis fields in the VAED—–this was necessary as intentional self-harm cannot be coded as a principal diagnosis.

### Independent variables

Patients were classified into three age groups (15–24, 25–64, > = 65 years), two gender groups (male and female), three geographic region groups (metropolitan, regional/rural and unknown/interstate/overseas) and four marital status groups (never married, married/de facto, divorced/separated/widowed and not stated). This information was sourced from the hospital admissions data. Socio economic status was classified as per the Socio-Economic Indexes for Areas (SEIFA) [23], based on the patient’s area of residence. The specific SEIFA used in this study was the Index of Relative Socio-Economic Advantage and Disadvantage; with state deciles based on local government areas; decile one indicates relatively the greatest disadvantage and least advantage.

### Statistical methods

Descriptive analysis of admissions linked to a suicide within one year was carried out using means, medians and proportions. The main measure used to assess the ‘risk’ of mortality within one year of hospital discharge in the study population was the Standardised Mortality Ratio (SMR). The SMRs compare the observed suicide mortality in the study population in each diagnosis group with the expected suicide mortality in the general population. Suicide mortality rates for the general population was computed using suicide data from the VSR and population data from the Australian Bureau of Statistics [24]. SMRs were calculated using the indirect method adjusting for age (5-year groups) and gender [25].

Survival analysis was carried out on conditions with high SMRs (SMR>5) to assess for competing risks due to non-suicidal deaths. Survival analysis was limited to index POCs. Those alive at one year were censored. Analysis was carried out with and without accounting for competing risks (i.e., death due to causes other than suicide) using the cumulative incidence function method as defined by Fine and Gray [26] and the Cox proportional hazards model, respectively. The independent variables age group, gender, marital status, geographic region and SEIFA were assessed for association with the outcome of suicide using a stepwise forward regression; all except SEIFA were significant to the outcome (assessed using the Akaike Information Criterion [27]). All tests were carried out at the 5% level of significance. SAS software, Version 9.4 [28] and Stata 16.0 (StataCorp) [29] was used to analyse the data.

The study was approved by the Monash University Human Research Ethics Committee (Project no: 14647). Historical administrative data was used. Name, date of birth and other identifiers were removed from the dataset by the data custodians prior to release of the data to the researchers. Due to the magnitude of the dataset, it was impractical to obtain consent.

## Results

### Overview of study population

A total of 1,737,743 patients with 2,430,154 hospital admissions during the study period 2011 to 2016 were selected for analysis; more than half of patients were aged 25–64 years (56.6%), and 60% were female. Seventy-one conditions related to injury, mental and physical illness were identified at the three-digit level of the ICD-10-AM from these hospital admission records.

### Conditions associated with at least five suicides

Forty-three of the 71 conditions were linked to at least five suicides in the 1-year follow-up period: these consisted of 14 physical illnesses, 5 symptoms, signs and abnormal clinical and laboratory findings, 12 mental health conditions, and 12 types of injury and poisonings. Selecting only hospital admissions with one of these 43 conditions appearing in the principal diagnosis field resulted in a sub-set of 756,007 admissions (by 538,910 patients) (Table 1). The most common mental health conditions were disorders due to the use of alcohol, and schizophrenia. The most common physical conditions were pneumonia, acute myocardial infarction and cellulitis. Among symptoms, signs and abnormal clinical and laboratory findings, pain in throat and chest, and abdominal and pelvic pain were the most common conditions. Among injury and poisonings, injuries to the head, and wrist and hand were most common.

**Table 1:** Hospital admissions for physical illnesses, mental health conditions and injury by age and gender, Victoria, 2011 to 2016.

Conditions (3-digit ICD-10 level)	No. of admissions^1^	n (%)			% female
		15–24 years	25–64 years	65+ years	
Certain infectious and parasitic diseases (A00-B99)					
A09 Other gastroenteritis and colitis of infectious and unspecified origin	36313	5982 (16.5)	19477 (53.6)	10854 (29.9)	62.8
Intermediate hyperglycaemia and diabetes mellitus (E09–E14)					
E11 Type 2 diabetes mellitus	11583	98 (0.8)	4839 (41.8)	6646 (57.4)	39.1
Mental and behavioural disorders (F00-F99)					
F10 Mental and behavioural disorders due to use of alcohol	11979	3022 (25.2)	7819 (65.3)	1138 (9.5)	37.0
F12 Mental and behavioural disorders due to use of cannabinoids	1221	## (##)	719 (58.9)	## (##)	27.7
F15 Mental and behavioural disorders due to use of other stimulants, including caffeine	2664	## (##)	1904 (71.5)	# (#)	31.6
F20 Schizophrenia	10169	859 (8.4)	8945 (88.0)	365 (3.6)	32.0
F23 Acute and transient psychotic disorders	1543	335 (21.7)	1113 (72.1)	95 (6.2)	46.7
F25 Schizoaffective disorders	3530	237 (6.7)	3168 (89.7)	125 (3.5)	48.5
F29 Unspecified nonorganic psychosis	2720	827 (30.4)	1787 (65.7)	106 (3.9)	42.0
F31 Bipolar affective disorder	4775	480 (10.1)	3987 (83.5)	308 (6.5)	56.0
F32 Depressive episode	9380	2195 (23.4)	6078 (64.8)	1107 (11.8)	53.4
F41 Other anxiety disorders	5453	898 (16.5)	3272 (60.0)	1283 (23.5)	65.9
F43 Reaction to severe stress, and adjustment disorders	5865	1366 (23.3)	4294 (73.2)	205 (3.5)	49.7
F60 Specific personality disorders	2533	878 (34.7)	1631 (64.4)	24 (0.9)	69.8
Diseases of the circulatory system (I00-I99)					
I21 Acute myocardial infarction	37804	18 (0.0)	16307 (43.1)	21479 (56.8)	31.5
I48 Atrial fibrillation and flutter	30853	185 (0.6)	10333 (33.5)	20335 (65.9)	50.3
I50 Heart failure	26042	23 (0.1)	2915 (11.2)	23104 (88.7)	49.4
Influenza and pneumonia (J09–J18)					
J18 Pneumonia, organism unspecified	47555	1436 (3.0)	16419 (34.5)	29700 (62.5)	47.5
J44 Other chronic obstructive pulmonary disease	31474	13 (0.0)	7154 (22.7)	24307 (77.2)	48.2
Diseases of the digestive system (K00-K93)					
K29 Gastritis and duodenitis	11680	1810 (15.5)	7511 (64.3)	2359 (20.2)	57.3
K56 Paralytic ileus and intestinal obstruction without hernia	14921	335 (2.2)	6130 (41.1)	8456 (56.7)	52.9
K59 Other functional intestinal disorders	12669	1016 (8.0)	4630 (36.5)	7023 (55.4)	56.8
K80 Cholelithiasis	24881	1618 (6.5)	15907 (63.9)	7356 (29.6)	63.6
Diseases of the skin and subcutaneous tissue (L00-L99)					
L03 Cellulitis	36685	2735 (7.5)	20516 (55.9)	13434 (36.6)	41.9
Other dorsopathies (M50–M54)					
M54 Dorsalgia	29327	1279 (4.4)	17587 (60.0)	10461 (35.7)	57.6
Diseases of the genitourinary system (N00-N99)					
N13 Obstructive and reflux uropathy	13875	465 (3.4)	10031 (72.3)	3379 (24.4)	28.3
Symptoms, signs and abnormal clinical and laboratory findings, NEC (R00-R99)					
R07 Pain in throat and chest	126012	3447 (2.7)	82074 (65.1)	40491 (32.1)	50.2
R10 Abdominal and pelvic pain	68291	14825 (21.7)	41639 (61.0)	11827 (17.3)	70.0
R41 Other symptoms and signs involving cognitive functions and awareness	4885	180 (3.7)	1252 (25.6)	3453 (70.7)	52.9
R45 Symptoms and signs involving emotional state	2823	900 (31.9)	1768 (62.6)	155 (5.5)	48.1
R55 Syncope and collapse	32642	2105 (6.4)	10309 (31.6)	20228 (62.0)	53.3
Injury, poisoning and certain other consequences of external causes (S00-T98)					
S01 Open wound of head	17262	2362 (13.7)	6827 (39.5)	8073 (46.8)	41.9
S06 Intracranial injury	16469	4180 (25.4)	7111 (43.2)	5178 (31.4)	35.8
S09 Other and unspecified injuries of head	7612	1750 (23.0)	3049 (40.1)	2813 (37.0)	49.8
S22 Fracture of rib(s), sternum and thoracic spine	12788	717 (5.6)	5845 (45.7)	6226 (48.7)	41.9
S51 Open wound of forearm	3736	694 (18.6)	1776 (47.5)	1266 (33.9)	36.5
S61 Open wound of wrist and hand	15170	3153 (20.8)	10049 (66.2)	1968 (13.0)	25.9
T38 Poisoning by hormones and their synthetic substitutes and antagonists, not elsewhere classified	764	94 (12.3)	407 (53.3)	263 (34.4)	53.7
T39 Poisoning by nonopioid analgesics, antipyretics and antirheumatics	5275	2745 (52.0)	2322 (44.0)	208 (3.9)	75.4
T40 Poisoning by narcotics and psychodysleptics [hallucinogens]	2529	445 (17.6)	1882 (74.4)	202 (8.0)	43.5
T42 Poisoning by antiepileptic, sedative-hypnotic and antiparkinsonism drugs	5737	1024 (17.8)	4128 (72.0)	585 (10.2)	62.8
T43 Poisoning by psychotropic drugs, not elsewhere classified	6244	2221 (35.6)	3809 (61.0)	214 (3.4)	63.2
T58 Toxic effect of carbon monoxide	274	30 (10.9)	219 (79.9)	25 (9.1)	24.1
Total (of 43 conditions)	756007	70232 (9.3)	388939 (51.4)	296836 (39.3)	50.4
Total (of all conditions)	2430154	263735 (10.9)	1375577 (56.6)	790842 (32.5)	60.1

Note: Includes conditions which led to at least five suicides within one year of discharge from hospital

# Cells < 5 suppressed to maintain confidentiality. ## Cells in the same row/columns suppressed to avoid recalculation of small cells

1. Subsequent admissions selected if they occurred one year after previous admission.

### Suicides

Over the five-year study period, 756,007 hospital admissions related to the selected 43 conditions (conditions with at least five suicides) were associated with a total of 631 suicides within one year of hospital discharge (Table 2). The mean and median ages of those who died by suicide following hospital admission for the selected 43 conditions were approximately 44 and 43 years respectively, and males accounted for 67.2%.

**Table 2:** Number of hospital admissions, suicides, SMRs and patient demographics for death by suicide within one year of hospital discharge (conditions with > = 5 suicides) Victoria, 2011 to 2016.

Conditions (3-digit ICD-10 level)	No. of admissions^1^	No. of suicides at one year	SMR (95% CI)	Mean (and median) age at suicide	% female
Certain infectious and parasitic diseases (A00-B99)					
A09 Other gastroenteritis and colitis of infectious and unspecified origin	36313	6	1.5 (0.6–3.1)	64.0 (53.5)	#
*A099 Gastroenteritis and colitis of unspecified origin*	*30992*	*6*			
Intermediate hyperglycaemia and diabetes mellitus (E09–E14)					
E11 Type 2 diabetes mellitus	11583	5	2.9 (1.1–6.5)	48.2 (43.0)	#
Mental and behavioural disorders (F00-F99)					
F10 Mental and behavioural disorders due to use of alcohol	11979	27	15.5 (10.4–22.3)	45.9 (44.0)	40.7
*F100 Acute intoxication*	*7947*	*14*			
*F101 Harmful use*	*658*	*5*			
F12 Mental and behavioural disorders due to use of cannabinoids	1221	6	33.3 (13.5–69.3)	29.3 (31.5)	#
*F125 Psychotic disorder*	*654*	*5*			
F15 Mental and behavioural disorders due to use of other stimulants, including caffeine	2664	12	30.0 (16.3–51.0)	31.8 (29.5)	#
*F155 Psychotic disorder*	*1438*	*5*			
F20 Schizophrenia	10169	37	22.2 (15.8–30.2)	38.2 (39.0)	5.4
*F200 Paranoid schizophrenia*	*2830*	*9*			
*F209 Schizophrenia*, *unspecified*	*6371*	*28*			
F23 Acute and transient psychotic disorders	1543	12	57.1 (31.0–97.2)	32.2 (29.0)	#
*F239 Unspecified*	*1203*	*11*			
F25 Schizoaffective disorders	3530	16	32.0 (18.9–50.9)	38.9 (33.5)	37.5
*F259 Unspecified*	*2465*	*12*			
F29 Unspecified nonorganic psychosis	2720	14	37.8 (21.5–62.0)	38.9 (39.0)	57.1
F31 Bipolar affective disorder	4775	12	19.4 (10.5–32.9)	43.1 (44.5)	#
*F319 Unspecified*		5			
$F32 Depressive episode	9380	86	72.9 (58.7–89.6)	41.8 (43.5)	36.0
*F322 Severe depressive episode without psychotic symptoms*	*2389*	*30*			
*F323 Severe depressive episode with psychotic symptoms*	*959*	*6*			
*F329 Depressive episode*, *unspecified*	*5336*	*49*			
F41 Other anxiety disorders	5453	20	33.3 (20.9–50.6)	45.4 (46.5)	60.0
*F412 Mixed anxiety and depressive disorder*	*909*	*8*			
*F419 Anxiety disorder*, *unspecified*	*3524*	*8*			
$F43 Reaction to severe stress, and adjustment disorders	5865	32	41.0 (28.5–57.2)	39.8 (41.5)	28.1
*F430 Acute stress reaction*	*1618*	*7*			
*F432 Adjustment disorders*	*3244*	*23*			
F60 Specific personality disorders	2533	17	65.4 (39.4–102.6)	36.2 (35.0)	47.1
*F603 Emotionally unstable personality disorder*	*2012*	*13*			
Diseases of the circulatory system (I00-I99)					
I21 Acute myocardial infarction	37804	6	1.0 (0.4–2.1)	78.7 (81.0)	#
*I214 Acute subendocardial myocardial infarction*		*5*			
I48 Atrial fibrillation and flutter	30853	8	2.0 (0.9–3.9)	68.0 (63.0)	0.0
I50 Heart failure	26042	6	1.6 (0.6–3.3)	79.5 (82.5)	#
*I500 Congestive heart failure*	*22220*	*5*			
Influenza and pneumonia (J09–J18)					
J18 Pneumonia, organism unspecified	47555	5	0.7 (0.3–1.6)	59.4 (62.0)	#
*J189 Pneumonia*, *unspecified*	*44965*	*5*			
J44 Other chronic obstructive pulmonary disease	31474	7	1.7 (0.7–3.3)	73.4 (76.0)	#
*J440 Chronic obstructive pulmonary disease with acute lower respiratory infection*	*22679*	*6*			
Diseases of the digestive system (K00-K93)					
K29 Gastritis and duodenitis	11680	7	4.9 (2.2–9.8)	52.9 (50.0)	#
K56 Paralytic ileus and intestinal obstruction without hernia	14921	5	2.6 (1.0–5.8)	77.2 (84.0)	#
K59 Other functional intestinal disorders	12669	5	3.1 (1.1–6.9)	77.6 (81.0)	0.0
*K590 Constipation*	*12257*	*5*			
K80 Cholelithiasis	24881	5	1.7 (0.6–3.8)	47.8 (49.0)	0.0
Diseases of the skin and subcutaneous tissue (L00-L99)					
L03 Cellulitis	36685	9	1.7 (0.8–3.1)	47.3 (48.0)	#
*L031 Cellulitis of other parts of limb*	*28752*	*8*			
Other dorsopathies (M50–M54)					
M54 Dorsalgia	29327	6	1.6 (0.7–3.4)	50.5 (49.0)	#
Diseases of the genitourinary system (N00-N99)					
N13 Obstructive and reflux uropathy	13875	5	2.2 (0.8–4.8)	41.4 (36.0)	0.0
Symptoms, signs and abnormal clinical and laboratory findings, NEC (R00-R99)					
$R07 Pain in throat and chest	126012	31	1.8 (1.3–2.5)	52.4 (51.0)	22.6
*R073 Other chest pain*	*24042*	*8*			
*R074 Chest pain*, *unspecified*	*100756*	*23*			
R10 Abdominal and pelvic pain	68291	13	1.8 (1.0–3.1)	41.8 (39.0)	#
*R101 Pain localised to upper abdomen*	*18601*	*6*			
*R104 Other and unspecified abdominal pain*	*29003*	*5*			
R41 Other symptoms and signs involving cognitive functions and awareness	*4885*	6	9.0 (3.6–18.6)	47.0 (47.5)	#
R45 Symptoms and signs involving emotional state	2823	16	43.2 (25.6–68.7)	41.2 (37.0)	#
*R458 Other symptoms and signs involving emotional state*	*2489*	*15*			
R55 Syncope and collapse	32642	6	1.4 (0.6–2.9)	61.3 (60.5)	#
Injury, poisoning and certain other consequences of external causes (S00-T98)					
S01 Open wound of head	17262	5	2.0 (0.7–4.3)	31.6 (28.0)	0.0
S06 Intracranial injury	16469	13	5.5 (3.1–9.2)	38.8 (39.0)	#
*S060 Concussive injury*	*8999*	*6*			
S09 Other and unspecified injuries of head	7612	5	5.1 (1.9–11.3)	41.4 (41.0)	#
S22 Fracture of rib(s), sternum and thoracic spine	12788	5	2.7 (1.0–5.9)	44.4 (37.0)	#
S51 Open wound of forearm	3736	6	10.3 (4.2–21.5)	40.0 (32.5)	#
S61 Open wound of wrist and hand	15170	5	2.0 (0.7–4.5)	34.0 (26.0)	#
T38 Poisoning by hormones and their synthetic substitutes and antagonists, not elsewhere classified	764	8	80.0 (37.2–151.9)	54.8 (55.0)	#
*T383 Insulin and oral hypoglycaemic [antidiabetic] drugs*	*663*	*8*			
$T39 Poisoning by nonopioid analgesics, antipyretics and antirheumatics	5275	31	66.0 (45.6–92.5)	36.4 (30.0)	58.1
*T391 4-Aminophenol derivatives*	*4495*	*22*			
*T393 Other nonsteroidal anti-inflammatory drugs [NSAID]*	*636*	*7*			
T40 Poisoning by narcotics and psychodysleptics [hallucinogens]	2529	10	27.8 (14.1–49.5)	34.4 (32.0)	#
$T42 Poisoning by antiepileptic, sedative-hypnotic and antiparkinsonism drugs	5737	48	71.6 (53.4–94.2)	44.3 (45.5)	33.3
*T424 Benzodiazepines*	*4515*	*39*			
*T426 Other antiepileptic and sedative-hypnotic drugs*	*662*	*5*			
$T43 Poisoning by psychotropic drugs, not elsewhere classified	6244	40	58.0 (42.0–78.2)	37.4 (36.5)	50.0
*T430 Tricyclic and tetracyclic antidepressants*	*504*	*6*			
*T432 Other and unspecified antidepressants*	*2098*	*8*			
*T435 Other and unspecified antipsychotics and neuroleptics*	*2147*	*17*			
*T436 Psychostimulants with potential for use disorder*	*1055*	*6*			
T58 Toxic effect of carbon monoxide	274	7	140.0 (61.2–276.9)	40.1 (39.0)	0.0
Total	756007	631		43.9 (43.0)	32.8

Note: Includes conditions which led to at least five suicides within one year of discharge from hospital

# Cells < 5 suppressed to maintain confidentiality.

$ Conditions with >30 suicides

1. Subsequent admissions selected if they occurred one year after previous admission.

Schizophrenia, depressive episodes, reaction to severe stress and adjustment disorders, throat and chest pains, nonopioid poisonings, poisoning by antiepileptic, sedative-hypnotic and antiparkinsonism drugs and poisoning by psychotropic drugs were some of the conditions associated with the highest numbers of suicides (>30) during the follow-up period.

**Fig 1:**
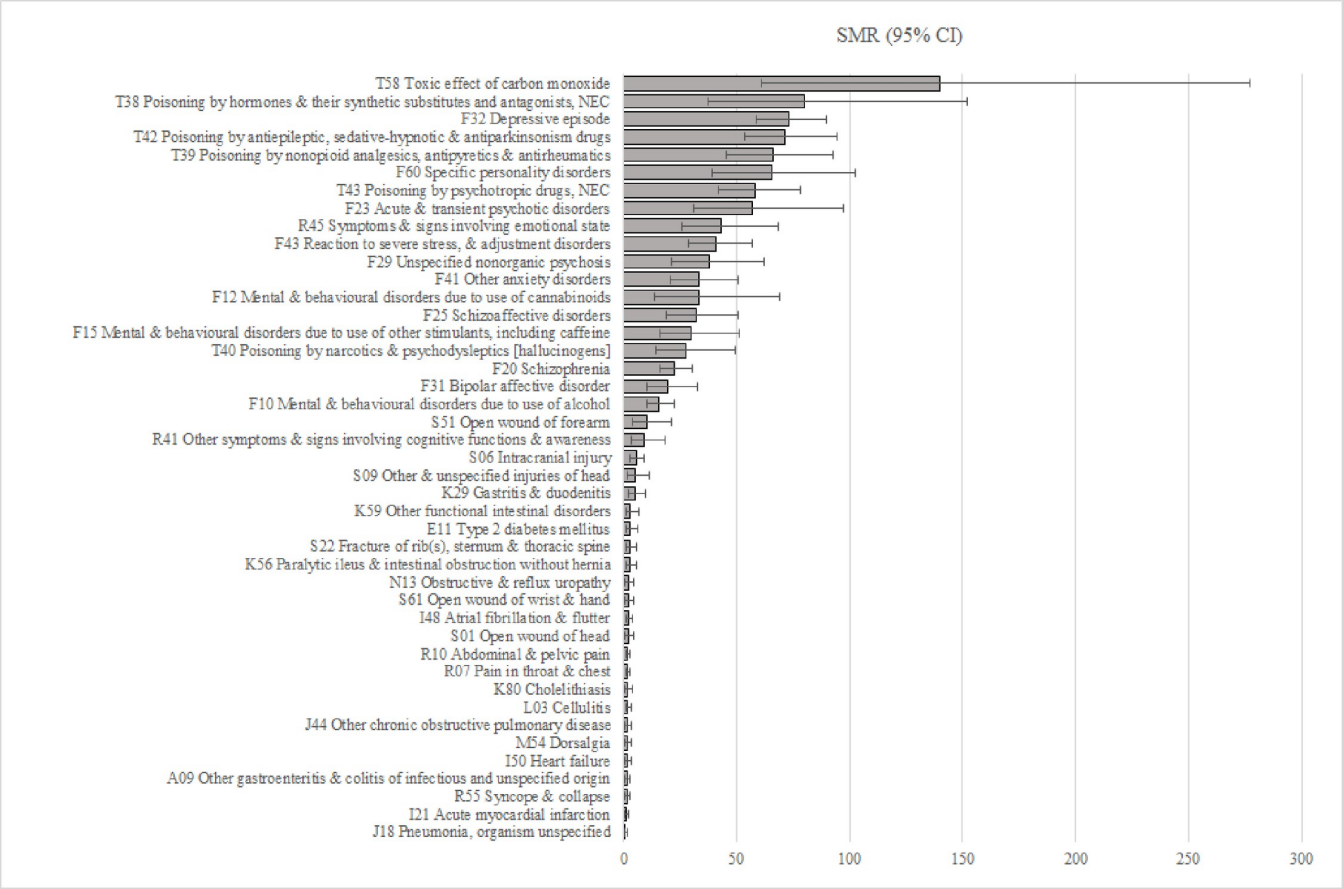
SMR (95% CI) for forty-three conditions associated with at least five suicides.

SMRs were calculated for each of the 43 conditions: poisonings had the highest SMRs ranging from 27.8 (CI 14.1–49.5) for poisoning by narcotics and psychodysleptics to 140 (CI 61.2–276.9) for carbon monoxide poisoning; 81% of these were intentional-self-harm incidents. SMRs related to mental and behavioural disorders followed, ranging from 15.5 (CI 10.4–22.3) for mental and behavioural disorders due to use of alcohol to 72.9 (CI 58.7–89.6) for depressive episodes (Fig 1). Admissions with a diagnosis code indicating depressive episodes, that also had external cause codes indicating intentional self-harm, had double the SMR (SMR = 150.0 (95% CI 38.2–408.2)) compared to admissions with depressive episodes overall (SMR = 72.9 (95% CI 58.7–89.6)) (not shown in tables). Of the physical illnesses, gastritis had the highest SMR of 4.9 (CI 2.2–9.8). Among *Symptoms*, *signs and abnormal clinical and laboratory findings*, admissions related to *Symptoms and signs involving emotional state* had a high SMR of 43.2 (CI 25.6–68.7) with 50% of these coded to *suicidal ideation*.

### Intentional self-harm

Out of the 43 selected conditions (conditions with > = 5 suicides), external cause codes were particularly relevant to poisoning-related admissions, which were mostly coded as caused by intentional self-harm, and intracranial injuries, which were mostly coded as caused by assaults (not shown in tables). Since intentional self-harm is known to be associated with suicide, self-harm related admissions were selected and further analysed. One-hundred and sixty-seven suicides were associated with intentional self-harm related admissions in the year prior to suicide (Table 3). It is important to note that there is an overlap with the numbers presented in Tables 2 and 3; i.e., of the 167 suicides presented in Table 3, the majority (n = 127) are also included in Table 2. The mean and median age of those who died by suicide following self-harm related hospital admission was approximately 41 years, and 41.3% of those who died were female.

**Table 3:** Hospital-admissions for intentional self-harm selected using external cause information, Victoria, 2011 to 2016.

No. of admissions^1^	19157
15–24 years, n (%)	6378 (33.3)
25–64 years, n (%)	11726 (61.2)
65+ years, n (%)	1053 (5.5)
Female, %	63.9
Suicides at one year	167
SMR (95% CI)	78.8 (67.5–91.4)
Age at suicide, mean (median)	41.5 (41.0)
Female, %	41.3

Note: 1. Cases selected if codes "X60" to "X84" (intentional self-harm) appeared in any of the forty diagnosis fields.

### Death by other causes

Death due to other causes pose a competing risk for suicide. To ascertain the impact of competing risks, survival analysis was carried out for 23 conditions (those with an SMR > = 5). The baseline variables considered to have an association with increased hazard of suicide death were age-group, gender, marital status and geographic region. Due to the small number of suicides associated with most conditions, statistically reliable conclusions could not be drawn for most of them. Proportions of death by other causes, and details of the survival analysis is provided in the Table A and B in S1 File. Overall, adjusting for competing risks had no significant impact on the estimated likelihood of suicide for any of the conditions.

Statistical model outputs can be found in the S2 File.

## Discussion

This study aimed to assess the risk of suicide following hospital admission for mental health conditions, physical illnesses, injury and poisoning and specifically for intentional self-harm. Standardised Mortality Ratios were used to assess the risk of suicide for each health condition following hospital-discharge relative to the age- and sex-matched suicide risk in the general population. Poisonings (including those resulting from intentional self-harm) had some of the highest SMRs, followed by mental health conditions. These were followed by symptoms and signs and abnormal clinical and laboratory findings involving cognitive functions and emotional states, while the lowest SMRs were for physical illnesses. Age, gender, marital status and geographic region of residence were confounders that were associated with selected conditions as well as the outcome of suicide.

The risk of suicide varied with the *type* of condition. Separation from hospital for treatment of a mental health condition was associated with a greater risk of suicide (high SMR) within the 1-year follow-up period than for a physical illness. This was expected given previous research has found many mental health conditions to be associated with suicide [30–32]. Similarly, injury and poisoning related hospital admissions also showed a greater risk of suicide in the 1-year follow-up period after discharge when compared to physical illnesses. Again, this is expected given that many of the injury and poisoning admissions were a result of intentional self-harm and there is a known link between suicide attempts and subsequent suicide [12–14]. This study found little, and in some instances no, increased risk of suicide for most of the selected physical illnesses; a similar finding was reported by Roberts, John, Kandalama, Williams, Lyons and Lloyd in a study of similar design [9] in the United Kingdom. Therefore, hospital admission for certain mental health conditions and injuries (in particular poisonings) are still priority groups for interventions; these could be focussed on high-risk groups identified at hospital discharge.

Mental health conditions are a known risk factor for suicide. Certain mental health conditions found to have high SMRs in this study were similar to those found to increase suicide risk in a recent meta-review [30]; these were substance use and schizophrenia, depressive episodes, personality disorders, and bipolar disorders. Further two other studies also found high relative risks for conditions such as depressive disorders, psychotic disorders, mood disorders, personality disorders, substance use disorders, and anxiety disorders [31, 32]. In the current study, *suicidal ideation* was also found as a secondary diagnosis among many of the admissions with a principal diagnosis of a mental health condition. Suicidal ideation and tendencies (ICD-10-AM code R45.81) are generally coded under *Symptoms and signs involving emotional state* in the ICD-10-AM (but excludes Signs and symptoms constituting part of a mental disorder) as a principal diagnosis. Mental health conditions could sometimes be masked and appear as Symptoms and signs involving emotional state, which could explain why symptoms and signs involving emotional state showed high SMRs in this study.

Certain types of injuries, including poisonings, are known to be risk factors for suicide. Certain poisonings observed in this study had high SMRs, indicating a high risk of subsequent suicide associated with discharge from hospital following a poisoning-related admission. This is expected as 81% of these poisoning cases resulted from intentional self-harm rather than unintentional incidents. Admissions for poisonings from benzodiazepines, analgesics and antipyretics such as 4-aminopheno derivatives (which commonly include paracetamol), antidepressants and antipsychotics were common among those who died by suicide within 1 year. This study confirms that hospital-admitted poisonings can be linked to subsequent suicide, and provides details regarding the types of poisonings that are associated with suicide, using Victorian hospital admissions data. The current study found carbon monoxide poisoning to have a high SMR. The Victorian government’s assertive outreach trials (HOPE program) target people treated in hospital for suicide attempts. People who have engaged in a suicide attempt using a method that was shown to have particularly high suicide risk in this study, for example carbon monoxide poisoning should be strongly encouraged to participate in these HOPE trials; people who present after carbon monoxide poisoning should be a priority for prevention. This has also been shown in a previous study by Hampson, Rudd and Hauff [33].

Among injuries other than poisonings, those with high SMRs were intracranial injuries (mostly concussive injury), other head injuries and open wounds of forearm. Open wounds of the forearm are likely indicative of intentional self- harm from cutting, which is a known risk factor for subsequent suicide [34]. This once again provides further support for known links between self-harm and suicide.

Certain physical illnesses are known to be associated with suicide. The physical illnesses with an SMR > 3 in this study were gastritis (some of which were alcohol related), constipation, and type 2 diabetes mellitus (SMR just under 3); the same three illnesses were found to have the highest SMRs in a study of similar design conducted by Roberts, John, Kandalama, Williams, Lyons and Lloyd [9] in the United Kingdom. Roberts, John, Kandalama, Williams, Lyons and Lloyd discussed that gastrointestinal diseases were often alcohol related (also seen in the current study) and constipation could be a side-effect of certain medications (e.g., opioids; constipation is also a known adverse effect for some pharmaceuticals to treat epilepsy, anaemia, pain, depression and other mental disorders). They noted that suicide risks were also linked to therapeutic medications such as those used for epilepsy and Parkinson’s disease (also seen in the current study). They noted that endocrine disorders that relate to poor blood glucose control (i.e. potentially indicative of poor chronic disease management) were also linked with suicide risk (also seen in the current study).

### Strengths

This study was drawn from state-wide population-level databases, providing reliable estimates of suicide risk following discharge from hospital for a range of physical and mental conditions. Data linkage provided appropriate follow-up. It also provided estimates intrinsic to Victoria, where the physical conditions identified as potential predictors of subsequent suicides were slightly different to what was seen elsewhere in the world (e.g., England and Wales [9]). Locally relevant data is key to informing locally applicable interventions. The competing risk modelling carried out in this study has demonstrated that death due to other causes is not a significant competing risk for suicide.

### Limitations

The SMRs computed in this study are limited to incidents serious enough to warrant hospital admission and do not include incidents that did not result in hospital admission.

Sample size was also a study limitation: larger samples lead to more comprehensive lists of conditions (48 conditions), as shown by Roberts, John, Kandalama, Williams, Lyons and Lloyd [9]. Comparatively, the current study consisted of a much smaller sample of admissions; however, 43 conditions were captured.

Selection of a condition is based on the fact that it was associated with at least five suicides. This could occur due to two reasons. The first is that the condition may be common (e.g., throat and chest pain); common conditions are therefore selected not because of a *disproportionate risk of suicide*, but because the overall case numbers are high, and eventually five suicides are likely to be observed. The second is that the condition can be associated with increased suicide risk. These underlying reasons for inclusion of conditions should be considered when drawing conclusions from this study.

The current study limited the selection of cases to those with an unplanned admission. This may result in loss of information on conditions that require ongoing hospital monitoring (e.g., kidney patients on dialysis). Furthermore, suicide risk among those with unplanned admissions may be different to suicide risk among those with planned admissions. Suicide risk among planned admissions is beyond the scope of this study. Future work in this area can expand the study scope and also include specific illnesses and treatments that fall within this category and retaining planned admissions.

Modelling techniques require a certain number of cases with the outcome (i.e., suicide, in this study) in order to produce reliable estimates of association between exposure variables and outcomes. Suicide is a rare event. In this study, for many of the physical or mental health conditions, there were insufficient numbers of cases with a suicide outcome to provide reliable estimates for the survival analysis that was performed. In interpreting the results, it should be noted that the absence of a statistically significant result does not necessarily demonstrate the absence of a correlation: for some conditions, case numbers were insufficient for statistical testing. Replicating this study in a larger population (e.g. a national study) would help to address this study limitation.

## Conclusions

Specified mental health conditions and types of injury and poisonings can be prioritised for targeting suicide prevention initiatives for persons discharged from hospitals: hospitals can therefore serve as a service contact point as well as a suicide risk identification platform. Among mental health conditions, priority can be given to depressive episodes, followed by personality disorders and psychotic episodes. Among poisonings, priority can be given to poisoning by antiepileptic, sedative-hypnotic and antiparkinsonism drugs, poisoning by nonopioid analgesics, antipyretics and antirheumatics, poisoning by unclassified psychotropic drugs, carbon monoxide poisoning and poisoning by hormone pharmaceuticals such as insulin. Intentional self-harm is an injury type that can also be prioritised. Staff working in clinical mental health services and emergency departments, could be advised of the findings relating to high suicide risk associated with particular methods or conditions, to assist with targeted suicide prevention.

## Supporting information

S1 File Deaths by other causes and survival analysis. (DOCX)

S2 File Statistical model outputs. (DOCX)
