# Supplementary material for: Suicide following hospital admission for mental health conditions, physical illness, injury and intentional self-harm in Victoria, Australia
Source: PLoS One. 2022 Jul 11;17(7):e0271341. doi: 10.1371/journal.pone.0271341 (PMC9273064; doi:10.1371/journal.pone.0271341)
Supplement: S1 File — (DOCX) [file pone.0271341.s001.docx]

# Supporting Information File

## Death by other causes

Deaths due to other reasons (apart from suicide) were captured in the VDI. The aim of this component of the analysis was to ascertain if there was a significant competing risk posed by deaths due to other causes, on the outcome of suicide. A competing risk is imposed if a patient dies due to another cause, which then eliminates the chance for a death to occur due to suicide. Table A presents socio-demographics and disease/injury profiles of these who died by suicide vs. other causes. For 23 conditions (those with SMRs > 5, considering only index admissions), approximately 0.39% died by suicide compared to 2.04% due to other causes. Suicide deaths were *least* common (0.14%) in the age group 65+ years whereas other deaths were *most* common (10.44%) in this age group. Suicide deaths were more common among males than females, whereas the occurrence of other deaths were similar among the two genders. Suicides and other deaths were most common among those with marital status ‘separated/divorced/widowed’. The proportion of suicide deaths and other deaths were equal among those living in regional/rural Victoria vs. those living in metropolitan Melbourne.

Suicide deaths were most common among patients admitted for: toxic effects of carbon monoxide (2.64%); depressive episodes (0.99%); poisoning by antiepileptic, sedative-hypnotic and antiparkinsonism drugs (0.81%); acute and transient psychotic disorders (0.8%); and specific personality disorders (0.79%). Other deaths were most common among patients admitted for: other symptoms and signs involving cognitive functions and awareness (12.65%); open wound of forearm (3.78%); other and unspecified injuries of head (3.37%); intracranial injury (3.16%); toxic effect of carbon monoxide (3.20%); and poisoning by narcotics and psychodysleptics [hallucinogens] (3.03%). Suicide deaths were more common than other deaths among patients admitted for self-harm.

**Table A: Proportion of deaths due to suicide and non-suicide for 23 conditions (SMR>5) (index admissions only), Victoria 2011-2016**

|  | Died by suicide % | Died of other causes % |
| --- | --- | --- |
| n (%)(N=81569) | 322 (0.39) | 1664 (2.04) |
| *Variable* |  |  |
| Age |  |  |
| 15-24 years | 0.26 | 0.13 |
| 25-64 years | 0.52 | 0.79 |
| 65+ years | 0.14 | 10.44 |
| Gender |  |  |
| Male | 0.49 | 2.01 |
| Female | 0.28 | 2.07 |
| Marital status |  |  |
| Never married | 0.40 | 0.73 |
| Married/defacto | 0.34 | 2.53 |
| Separated/divorced/widowed | 0.51 | 6.44 |
| Not stated | 0.32 | 2.03 |
| Geographical region |  |  |
| Melbourne Metropolitan Area | 0.40 | 2.06 |
| Regional/Rural Victoria | 0.43 | 2.23 |
| Interstate/Overseas/Unknown | 0.10 | 0.76 |
| Condition |  |  |
| F10 Mental and behavioural disorders due to use of alcohol | 0.13 | 1.36 |
| F12 Mental and behavioural disorders due to use of cannabinoids | 0.34 | 0.69 |
| F15 Mental and behavioural disorders due to use of other stimulants, including caffeine | 0.31 | 0.55 |
| F20 Schizophrenia | 0.32 | 1.02 |
| # F23 Acute and transient psychotic disorders | 0.80 | 0.48 |
| F25 Schizoaffective disorders | 0.48 | 0.71 |
| F29 Unspecified nonorganic psychosis | 0.57 | 0.72 |
| F31 Bipolar affective disorder | 0.25 | 0.71 |
| # F32 Depressive episode | 0.99 | 0.93 |
| F41 Other anxiety disorders | 0.31 | 1.26 |
| F43 Reaction to severe stress, and adjustment disorders | 0.70 | 0.45 |
| # F60 Specific personality disorders | 0.79 | 0.53 |
| $ R41 Other symptoms and signs involving cognitive functions and awareness | 0.06 | 12.65 |
| R45 Symptoms and signs involving emotional state | 0.48 | 1.19 |
| S06 Intracranial injury | 0.09 | 3.16 |
| $ S09 Other and unspecified injuries of head | 0.07 | 3.37 |
| $ S51 Open wound of forearm | 0.14 | 3.78 |
| T38 Poisoning by hormones and their synthetic substitutes and antagonists, not  elsewhere classified | 0.61 | 2.63 |
| T39 Poisoning by nonopioid analgesics, antipyretics and antirheumatics | 0.62 | 0.40 |
| $ T40 Poisoning by narcotics and psychodysleptics [hallucinogens] | 0.49 | 3.03 |
| # T42 Poisoning by antiepileptic, sedative-hypnotic and antiparkinsonism drugs | 0.81 | 1.25 |
| T43 Poisoning by psychotropic drugs, not elsewhere classified | 0.61 | 0.51 |
| #*T58 Toxic effect of carbon monoxide | 2.74 | 3.20 |
| Intentional self-harm | 0.91 | 0.74 |

Note: # Conditions with the highest proportions of suicide, $ conditions with the highest proportions of other deaths

## Survival analysis

A *patient outcomes* approach was used in the survival analysis: every patient could occur only once in the data. Twenty-three conditions (SMR>5) along with intentional self-harm were retained for survival analysis. These included all mental and behavioural disorders, some symptoms, signs and abnormal clinical and laboratory findings, and some injury and poisoning related hospital admissions.

The baseline variables considered to have an association with increased hazard of suicide death were age-group, gender, marital status and geographic region. Schizophrenia, depressive episodes, reaction to severe stress, and adjustment disorders, poisoning by nonopioid analgesics, antipyretics and antirheumatics, poisoning by narcotics and psychodysleptics [hallucinogens], poisoning by antiepileptic, sedative-hypnotic and antiparkinsonism drugs and poisoning by psychotropic drugs showed a statistical significance in the hazard of those with the condition compared to those without (Table B). Reliable conclusions could not be drawn for the remaining conditions due to low outcome numbers. Adjustment for age and gender decreased the hazard ratio for all conditions except for poisoning by nonopioid analgesics, antipyretics and antirheumatics (which increased). Adjustment for competing risks had no significant impact on the sub-hazard ratios for any of the conditions. Intentional self-harm and depressive episodes-related hospital admissions were indicative of some of the highest hazards of suicide.

**Table B: Survival analysis for conditions with SMR>5 (index admissions only)**

| Conditions (3-digit ICD-10 level) | SMR (95% CI) | Unadjusted hazard ratios (95% CI) | Age and sex adjusted hazard ratio (95%CI) | Sub-hazard ratio (SHR) (95% CI) | |
| --- | --- | --- | --- | --- | --- |
|  |  |  |  | Adjusted for age, sex and competing risk of death by non-suicide related causes | Adjusted for age, sex, marital status, geographic region, and competing risk of death by non-suicide related causes |
| Mental and behavioural disorders (F00-F99) |  |  |  |  |  |
| F10 Mental and behavioural disorders due  to use of alcohol | 15.5 (10.4-22.3) | 3.6 (2.0-6.6) | 2.5 (1.4-4.6) | 2.5 (1.4-4.6) | 2.0 (1.1-3.6) |
| F12 Mental and behavioural disorders due  to use of cannabinoids | 33.3 (13.5-69.3) | 9.6 (3.1-29.7) | 5.7 (1.8-17.7) | 5.7 (1.8-17.8) | 4.3 (1.4-13.5) |
| F15 Mental and behavioural disorders due  to use of other stimulants, including  caffeine | 30.0 (16.3-51.0) | 8.5 (3.5-20.5) | 5.1 (2.1-12.3) | 5.1 (2.1-12.3) | 3.8 (1.6-9.2) |
| #F20 Schizophrenia | 22.2 (15.8-30.2) | 8.9 (5.6-14.3) | 5.7 (3.5-9.1) | 5.7 (3.6-9.1) | 3.7 (2.3-6.0) |
| F23 Acute and transient psychotic disorders | 57.1 (31.0-97.2) | 22.6 (12.1-42.1) | 16.6 (8.9-31.0) | 16.7 (8.9-31.2) | 12.3 (6.5-23.0) |
| F25 Schizoaffective disorders | 32.0 (18.9-50.9) | 13.2 (6.6-26.6) | 9.8 (4.9-19.7) | 9.8 (4.9-19.8) | 6.4 (3.2-12.9) |
| F29 Unspecified nonorganic psychosis | 37.8 (21.5-62.0) | 16.2 (9.1-28.6) | 11.4 (6.4-20.2) | 11.4 (6.4-20.4) | 9.0 (5.0-16.2) |
| F31 Bipolar affective disorder | 19.4 (10.5-32.9) | 6.9 (3.3-14.5) | 5.6 (2.6-11.7) | 5.6 (2.7-11.8) | 4.0 (1.9-8.5) |
| #F32 Depressive episode | 72.9 (58.7-89.6) | 30.7 (23.8-39.5) | 25.3 (19.6-32.6) | 25.4 (19.6-32.9) | 20.4 (15.7-26.4) |
| F41 Other anxiety disorders | 33.3 (20.9-50.6) | 8.7 (4.9-15.3) | 8.6 (4.9-15.2) | 8.6 (4.9-15.3) | 7.4 (4.1-13.1) |
| #F43 Reaction to severe stress, and  adjustment disorders | 41.0 (28.5-57.2) | 20.0 (13.7-29.3) | 15.0 (10.2-21.9) | 15.0 (10.2-22.0) | 11.6 (7.9-17.1) |
| F60 Specific personality disorders | 65.4 (39.4-102.6) | 22.1 (11.5-42.7) | 20.1 (10.4-39.0) | 20.2 (10.4-39.1) | 14.8 (7.6-28.9) |
| Symptoms, signs and abnormal clinical and laboratory findings, NEC (R00-R99) |  |  |  |  |  |
| R41 Other symptoms and signs involving  cognitive functions and awareness | 9.0 (3.6-18.6) | 1.9 (0.5-7.5) | 2.2 (0.6-9.0) | 2.2 (0.5-8.9) | 1.9 (0.5-7.7) |
| R45 Symptoms and signs involving  emotional state | 43.2 (25.6-68.7) | 13.6 (7.0-26.3) | 10.2 (5.3-19.7) | 10.2 (5.3-19.8) | 8.0 (4.1-15.5) |
| Injury, poisoning and certain other consequences of external causes (S00-T98) |  |  |  |  |  |
| S06 Intracranial injury | 5.5 (3.1-9.2) | 2.6 (1.5-4.6) | 1.9 (1.1-3.3) | 1.9 (1.1-3.4) | 1.7 (0.9-3.0) |
| S09 Other and unspecified injuries of head | 5.1 (1.9-11.3) | 2.0 (0.8-5.4) | 1.7 (0.6-4.5) | 1.7 (0.6-4.5) | 1.5 (0.6-4.0) |
| S51 Open wound of forearm | 10.3 (4.2-21.5) | 4.0 (1.5-10.7) | 2.9 (1.1-7.9) | 3.0 (1.1-7.9) | 2.7 (1.0-7.2) |
| T38 Poisoning by hormones and their  synthetic substitutes and antagonists,  not elsewhere classified | 80.0 (37.2-151.9) | 16.9 (5.5-52.6) | 16.0 (5.1-49.5) | 16.0 (5.1-49.8) | 14.5 (4.6-45.1) |
| #T39 Poisoning by nonopioid analgesics,  antipyretics and antirheumatics | 66.0 (45.6-92.5) | 17.7 (11.9-26.4) | 20.2 (13.4-30.4) | 20.2 (13.3-30.7) | 16.8 (11.1-25.4) |
| #T40 Poisoning by narcotics and  psychodysleptics [hallucinogens] | 27.8 (14.1-49.5) | 13.7 (6.8-27.6) | 9.6 (4.8-19.4) | 9.6 (4.7-19.2) | 7.1 (3.5-14.4) |
| #T42 Poisoning by antiepileptic, sedative-  hypnotic and antiparkinsonism drugs | 71.6 (53.4-94.2) | 23.7 (16.7-33.7) | 21.9 (15.4-31.2) | 22.0 (15.4-31.3) | 17.2 (12.0-24.7) |
| #T43 Poisoning by psychotropic drugs, not  elsewhere classified | 58.0 (42.0-78.2) | 17.4 (11.7-25.8) | 15.7 (10.6-23.4) | 15.8 (10.6-23.6) | 12.2 (8.1-18.2) |
| T58 Toxic effect of carbon monoxide | 140.0 (61.2-276.9) | 78.1 (35.0-174.6) | 47.0 (21.0-105.1) | 47.0 (21.0-105.5) | 37.5 (16.7-84.0) |
| # Intentional self-harm | 78.8 (67.5-91.4) | 31.1 (25.6-37.9) | 30.3 (24.8-37.1) | 30.4 (24.7-37.6) | 24.3 (19.6-30.1) |

#Conditions with suicide numbers large enough to draw reliable conclusions
