# Supplementary material for: Suicide following hospital admission for mental health conditions, physical illness, injury and intentional self-harm in Victoria, Australia
Source: PLoS One. 2022 Jul 11;17(7):e0271341. doi: 10.1371/journal.pone.0271341 (PMC9273064; doi:10.1371/journal.pone.0271341)
Supplement: S2 File — (DOCX) [file pone.0271341.s002.docx]

Intentional self-harm unadjusted

*Unadjusted;

**proc** **phreg** data=suicide.VAED_Index_Survival ;

class ish/ref=first;

model time*event(**0**,**2**)=ish/rl;

**run**;

The SAS System 14:41 Friday, October 1, 2021 12

The PHREG Procedure

Testing Global Null Hypothesis: BETA=0

Test Chi-Square DF Pr > ChiSq

Likelihood Ratio 585.8650 1 <.0001

Score 2893.1051 1 <.0001

Wald 1173.0196 1 <.0001

Type 3 Tests

Wald

Effect DF Chi-Square Pr > ChiSq

ish 1 1173.0196 <.0001

Analysis of Maximum Likelihood Estimates

Parameter Standard Hazard 95% Hazard Ratio

Parameter DF Estimate Error Chi-Square Pr > ChiSq Ratio Confidence Limits Label

ish 1 1 3.43796 0.10038 1173.0196 <.0001 31.123 25.565 37.890 ish 1

*Intentional self-harm adjusted for age and sex, no competing risks;

**proc** **phreg** data=suicide.VAED_Index_Survival ;

class ish E_agegroup sex /ref=first;

model time*event(**0**,**2**)=E_agegroup sex ish/rl;

**run**;

The SAS System 14:41 Friday, October 1, 2021 14

The PHREG Procedure

Testing Global Null Hypothesis: BETA=0

Test Chi-Square DF Pr > ChiSq

Likelihood Ratio 865.8816 4 <.0001

Score 3176.3688 4 <.0001

Wald 1426.1718 4 <.0001

Type 3 Tests

Wald

Effect DF Chi-Square Pr > ChiSq

E_agegroup 2 38.9176 <.0001

sex 1 216.4748 <.0001

ish 1 1094.6199 <.0001

Analysis of Maximum Likelihood Estimates

Parameter Standard Hazard 95% Hazard Ratio

Parameter DF Estimate Error Chi-Square Pr > ChiSq Ratio Confidence Limits Label

E_agegroup 3 1 0.30491 0.11775 6.7057 0.0096 1.356 1.077 1.709 E_agegroup 3

E_agegroup 4 1 -0.37873 0.15135 6.2618 0.0123 0.685 0.509 0.921 E_agegroup 4

sex 2 1 -1.30123 0.08844 216.4748 <.0001 0.272 0.229 0.324 sex 2

ish 1 1 3.41178 0.10312 1094.6199 <.0001 30.319 24.771 37.110 ish 1

* Intentional self-harm adjusted for age and sex and competing risk;

**proc** **phreg** data=suicide.VAED_Index_Survival;

class ish E_agegroup sex/ order=internal ref=first param=glm;

model time*event(**0**) = E_agegroup sex ish / eventcode=**1**;

hazardratio 'Subdistribution Hazards' ish / diff=pairwise;

**run**;

The SAS System 14:41 Friday, October 1, 2021 16

The PHREG Procedure

Testing Global Null Hypothesis: BETA=0

Test Chi-Square DF Pr > ChiSq

Wald 1571.3927 4 <.0001

Type 3 Tests

Wald

Effect DF Chi-Square Pr > ChiSq

E_agegroup 2 43.9437 <.0001

sex 1 216.6381 <.0001

ish 1 1009.7685 <.0001

Analysis of Maximum Likelihood Estimates

Parameter Standard Hazard

Parameter DF Estimate Error Chi-Square Pr > ChiSq Ratio Label

E_agegroup 3 1 0.30028 0.12018 6.2426 0.0125 1.350 E_agegroup 3

E_agegroup 4 1 -0.43768 0.15738 7.7337 0.0054 0.646 E_agegroup 4

E_agegroup 2 0 0 . . . . E_agegroup 2

sex 2 1 -1.29745 0.08815 216.6381 <.0001 0.273 sex 2

sex 1 0 0 . . . . sex 1

ish 1 1 3.41562 0.10749 1009.7685 <.0001 30.436 ish 1

ish 0 0 0 . . . . ish 0

Subdistribution Hazards: Hazard Ratios for ish

Point 95% Wald Confidence

Description Estimate Limits

ish 1 vs 0 30.436 24.654 37.573

ish 0 vs 1 0.033 0.027 0.041

*Establishing the proper baseline model using forward progression and AIC statistic;

**proc** **phreg** data=suicide.VAED_Index_Survival ;

class E_agegroup sex newmarital GEOGRAPHIC_REGION sdlga/ ref=first;

model time*event(**0**)=E_agegroup sex newmarital GEOGRAPHIC_REGION sdlga/eventcode=**1**;

*hazardratio sex/diff=ref;

**run**;

The PHREG Procedure

Analysis of Maximum Likelihood Estimates

Parameter Standard

Parameter DF Estimate Error Chi-Square Pr > ChiSq

E_agegroup 3 1 0.53902 0.12317 19.1499 <.0001

E_agegroup 4 1 -0.42600 0.17252 6.0975 0.0135

sex 2 1 -1.17903 0.08953 173.4098 <.0001

newmarital 2 1 -1.08430 0.09725 124.3245 <.0001

newmarital 3 1 0.06659 0.13963 0.2274 0.6334

newmarital 4 1 0.20327 0.22400 0.8235 0.3642

GEOGRAPHIC_REGION 2 1 0.56598 0.12350 21.0027 <.0001

GEOGRAPHIC_REGION 3 1 -0.59305 0.29388 4.0722 0.0436

sdlga 2 1 -0.41126 0.22322 3.3944 0.0654

sdlga 3 1 -0.13121 0.21187 0.3835 0.5357

sdlga 4 1 -0.37221 0.21032 3.1318 0.0768

sdlga 5 1 -0.48379 0.22258 4.7244 0.0297

sdlga 6 1 -0.26252 0.19697 1.7764 0.1826

sdlga 7 1 -0.10289 0.18239 0.3183 0.5727

sdlga 8 1 0.14930 0.18376 0.6601 0.4165

sdlga 9 1 0.19561 0.17854 1.2004 0.2732

sdlga 10 1 -0.09453 0.19401 0.2374 0.6261

sdlga 99 0 0 . . .

Analysis of Maximum Likelihood Estimates

Hazard

Parameter Ratio Label

E_agegroup 3 1.714 E_agegroup 3

E_agegroup 4 0.653 E_agegroup 4

sex 2 0.308 sex 2

newmarital 2 0.338 newmarital 2

newmarital 3 1.069 newmarital 3

newmarital 4 1.225 newmarital 4

GEOGRAPHIC_REGION 2 1.761 Broad geographic region - based on LGAs 2

GEOGRAPHIC_REGION 3 0.553 Broad geographic region - based on LGAs 3

sdlga 2 0.663 sdlga 2

sdlga 3 0.877 sdlga 3

sdlga 4 0.689 sdlga 4

sdlga 5 0.616 sdlga 5

sdlga 6 0.769 sdlga 6

sdlga 7 0.902 sdlga 7

sdlga 8 1.161 sdlga 8

sdlga 9 1.216 sdlga 9

sdlga 10 0.910 sdlga 10

sdlga 99 . sdlga 99

*Final model;

**proc** **phreg** data=suicide.VAED_Index_Survival ;

class ish E_agegroup sex GEOGRAPHIC_REGION newmarital / ref=first;

model time*event(**0**)=ish E_agegroup sex GEOGRAPHIC_REGION newmarital /eventcode=**1**;

hazardratio ish/diff=ref;

**run**;

The SAS System 14:41 Friday, October 1, 2021 22

The PHREG Procedure

Analysis of Maximum Likelihood Estimates

Parameter Standard

Parameter DF Estimate Error Chi-Square Pr > ChiSq

GEOGRAPHIC_REGION 3 1 -0.50302 0.25540 3.8790 0.0489

newmarital 2 1 -0.91876 0.10048 83.6149 <.0001

newmarital 3 1 0.07677 0.13672 0.3153 0.5744

newmarital 4 1 0.09199 0.22327 0.1698 0.6803

Analysis of Maximum Likelihood Estimates

Hazard

Parameter Ratio Label

GEOGRAPHIC_REGION 3 0.605 Broad geographic region - based on LGAs 3

newmarital 2 0.399 newmarital 2

newmarital 3 1.080 newmarital 3

newmarital 4 1.096 newmarital 4

Hazard Ratios for ish

Point 95% Wald Confidence

Description Estimate Limits

ish 1 vs 0 24.255 19.554 30.087
